# Impact of rehabilitation with dental implants on the quality of life of patients undergoing maxillofacial reconstruction: a systematic review

**DOI:** 10.1007/s11136-024-03795-w

**Published:** 2024-10-17

**Authors:** Simra Azher, Roisin McGrath, Yasaman Mohammadi Kamalabadi, Georgios Tsakos, Felix Sim, Ankur Singh

**Affiliations:** 1 Melbourne Dental School. Faculty of Medicine, Dentistry and Health Sciences, University of Melbourne, Melbourne, VIC Australia; 2 Department of Dental Medicine and Oral Health Sciences, McGill University, Montreal, Canada; 3 Department of Epidemiology and Public Health, UCL, London, UK; 4 Melbourne School of Population and Global Health, Melbourne Dental School, University of Melbourne, Melbourne, VIC Australia

**Keywords:** Quality of life, Patient reported outcome measure (PROM), Content validity, Dental implant, Surgical flaps, Reconstruction

## Abstract

**Purpose:**

Maxillofacial reconstruction with dental implants in microvascular tissue flaps aims to improve mastication. However, the quality of life (QoL) impact of this intervention is yet to be determined. This systematic review assessed the QoL impact of maxillofacial reconstruction with implant-supported teeth compared to no dental rehabilitation, removable dentures, and obturator (modified denture). Additionally, we examined instruments applied to measure QoL in maxillofacial reconstruction.

**Methods:**

Databases Ovid Medline and Embase, Scopus, Web of Science and Handle on QoL were searched. Cohort, case–control and randomized controlled trials (RCT) were narratively synthesized for QoL captured through validated instruments. Study methodological quality was assessed using Cochrane Risk of Bias 2 and Risk of Bias in Non-randomized studies of Exposure. Instruments underwent COSMIN content validity analysis.

**Results:**

Of a total of 2735 studies screened, the three included studies (two cohort and one RCT) showed improved QoL with maxillofacial reconstruction compared to obturator and no dental rehabilitation. However, these studies have high risk of bias due to confounding. None of the instruments achieved a sufficient relevance rating for maxillofacial reconstruction, having been designed for other target populations and there is no evidence on their content validity for this population, but the EORTC QLQ30 H&N35 satisfied more COSMIN criteria than the UW-QOL and OHIP-14.

**Conclusion:**

Although studies showed favourable QoL with maxillofacial reconstruction involving dental implants, these have high risk of bias and further studies are needed to establish the impact. Existing QoL instruments lack content validity and tailored instruments are needed for QoL evaluation in maxillofacial reconstruction.

**Supplementary Information:**

The online version contains supplementary material available at 10.1007/s11136-024-03795-w.

## Introduction

Head and neck pathologies include benign tumours such as ameloblastoma and myxoma, osteoradionecrosis (ORN), which is radiation-associated necrosis of the jawbone, and malignant tumours including squamous cell carcinoma (SCC) [1–3]. These conditions are treated with resection surgery. Maxillofacial reconstruction aims to improve the quality of life (QoL) for patients with these conditions and involves restoration of the pathology defect. This is achieved with a microvascular free flap consisting of bone, muscle, fat, skin, and associated blood vessels harvested from distant body areas. Free flaps utilised in maxillo-mandibular reconstruction since the late 1980s are now the standard of care for jaw defects [4, 5]. Due to significant soft and hard tissue alterations with free flaps, traditional rehabilitation with removable dentures and obturators (modified denture) is at risk of compromised stability and poor sealing of oro-nasal cavities, potentially negatively impacting patient QoL [6]. Increasingly, maxillofacial reconstruction has incorporated dental implant-supported prosthetic teeth aimed at improving retention of the dental prosthesis and better restoration of function [7]. The QoL impact of this intervention compared to traditional interventions is yet to be determined.

Maxillofacial reconstruction has been shown to have positive QoL impacts [8–13]. However, some research documents no difference in QoL with maxillofacial reconstruction compared to traditional interventions [14]. Systematic reviews by Said et al. [15] and Wijbenja et al. [16] compared maxillofacial reconstruction against a single intervention, however excluded other well-known treatment comparators. Another review by Quadri et al. [17] focused on oral cancer populations, excluding reconstruction for benign pathology. Hence, a synthesis of all the evidence on maxillofacial reconstruction compared to traditional interventions (no dental rehabilitation, removable denture and obturator) is lacking.

A key consideration is how QoL is captured, as various instruments exist with different scoring algorithms [18]. Commonly used head and neck patient-reported outcome measure (PROM) instruments include the European Organization for Research and Treatment of Cancer Head and Neck 35 and Quality of life Questionnaire 30 (EORTC H&N 35 QLQ 30), the University of Washington QoL Scale (UWQOL), and the Oral Health Impact Profile-14 (OHIP-14) [19, 20]. Some of these instruments assess generic health-related QoL (HRQoL) and may therefore lack sensitivity to detect clinically important differences in patients undergoing maxillofacial reconstruction [21, 22]. On the other hand, specific oral health-related QoL (OHRQoL) instruments, such as the OHIP-14, may lack applicability beyond their intended focus, thereby limiting cross-intervention comparisons [23, 24]. It is unclear if existing instruments are appropriate for maxillofacial reconstruction, especially for QoL comparisons between interventions intended for multiple disease processes [25–30]. Therefore, an assessment of existing instruments is necessary to determine their relevance for maxillofacial reconstruction.

The aim of this systematic review was to synthesise the evidence on the QoL impacts of maxillofacial reconstruction with dental rehabilitation involving implants, as compared to traditional interventions following resection of benign or malignant pathology. This study also evaluated the measurement properties of instruments applied to measure QoL in patients undergoing maxillofacial reconstruction. For simplicity, the term QoL referring to HRQoL including OHRQoL will be used in this review. These terms are often used interchangeably in the literature, with various PROM instruments assessing one or both.

## Methods

The Preferred Reporting Items for Systematic Reviews and Meta-Analyses (PRISMA) guidelines were followed for this systematic review [31] and the protocol published on PROSPERO (ID: CRD42022364416).

### Review questions

1. What is the impact of maxillofacial reconstruction with dental implant rehabilitation following resection of pathology, on QoL, compared to other interventions (no dental rehabilitation, removable denture and obturator)?
2. Are the measurement properties of existing instruments applied to measure QoL in this population appropriate for maxillofacial reconstruction?

### Study inclusion criteria

1. Exposure/Intervention: maxillofacial reconstruction defined as bone or soft tissue microvascular free flap reconstruction plus dental rehabilitation involving implants (the implants support a dental prosthesis which may be fixed or removable)
2. Patients with benign or malignant pathology of the maxilla or mandible as per the International Classification of Diseases (ICD) classification [32]
3. Participants of any gender or geographical location
4. Traditional interventions used as a comparator are no dental rehabilitation, removable denture or obturator
5. Validated PROM instrument used for gathering QoL data
6. Randomized controlled trials (RCTs), cohort and case control studies
7. Any study publication date

### Study exclusion criteria

1. Head and neck pathology, including naso-pharyngeal tumors, not involving the maxilla or mandible
2. QoL data gathered through other means e.g. author made questionnaires or interviews
3. Language other than English

### Search strategy

Databases Ovid Medline and Embase, Scopus, Web of science and Handle on QoL were electronically searched. The search strategy was developed with the aid of University of Melbourne librarians by SA, with the process being overseen by AS and RM. Keywords/synonyms were identified for concepts “dental implant” and “quality of life” using literature searches and corresponding index terms in databases. Phrase searching was applied for multiple word terms. A search string was generated for each database (Ovid Medline and Embase combined) (Appendix 1a). Within the databases, searches were restricted to English language and human studies and duplicates were removed. Searches were conducted from 31st of January 2023 to 3rd February 2023 and updated on 5th of January 2024.

### Study selection

Search results were imported into Covidence Systematic Review Software and duplicates removed. Two authors (SA and YMK) independently performed title and abstract screening, followed by independent full text screening. Relevant study reference lists were manually searched for additional papers. The reason for exclusion was recorded. If information relevant to inclusion criteria was unavailable/unclear, respective authors were contacted for clarification. Conflicts at every stage were discussed between SA and YMK and if consensus could not be reached, a third reviewer (AS) was involved for resolution. The study design, whether reported or not in the primary publication, was verified independently by SA and YMK and any ambiguity was resolved in consultation with AS.

### Data extraction

Data were extracted into a predefined data collection form, which was piloted on two studies prior to data extraction. Data extraction was performed independently by two reviewers (SA and YMK) and any disagreements resolved by consultation with a third reviewer (AS). Data collected included author, year, country, study type, sample size, patient age range and gender. Disease characteristics such as pathology type (benign/malignant), ICD classification and defect type were extracted. Intervention characteristics such as flap type, implant number and placement timing, prosthesis type (fixed/removable) and insertion timing as well as comparator, radiation therapy, follow-up and instrument used were recorded. Finally, outcome measures including QoL effect estimate, uncertainty measures, sensitivity analysis of bias as well as study limitations as reported by respective authors were extracted.

### Assessment of methodological quality and synthesis

**Fig. 1:**
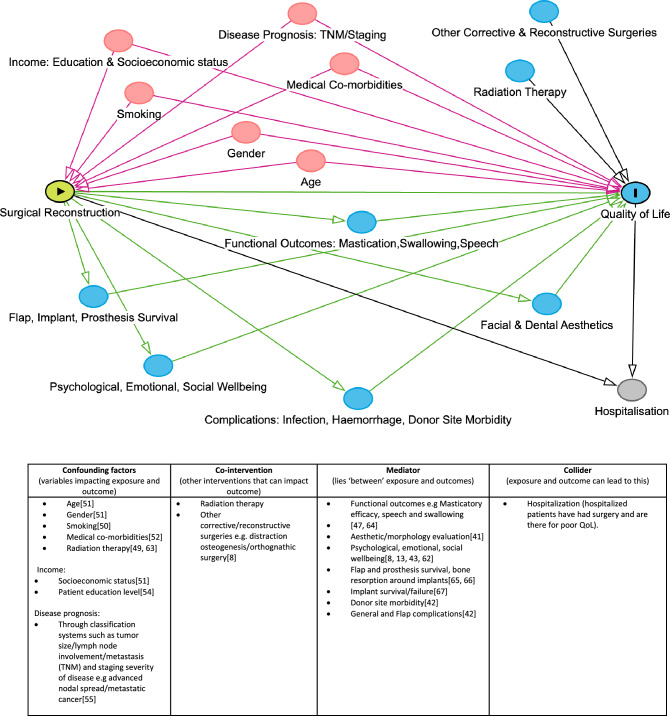
DAG Diagram

Study quality assessment was performed independently by two reviewers (SA and YMK) and any disagreements resolved by consensus with third reviewer (AS). Revised Cochrane Risk of Bias tool for randomized trials (RoB 2) examined studies across five domains for bias due to 1) randomization process, 2) deviations from intended interventions, 3) missing outcome data, 4) outcome measurement and 5) selection of reported result [33, 34]. Risk of Bias in Non-randomized Studies of Exposure (ROBINS E) tool for observational studies assessed seven domains for bias arising from 1) confounding, 2) measurement of exposure, 3) selection of participants, 4) post-exposure interventions, 5) missing data, 6) outcome measurement and 7) selection of reported results [35]. A Directed Acyclic Graph (DAG) was constructed as a preliminary step to the application of ROBINS E for identification of confounding factors, mediators and colliders against which included studies could be assessed (see Fig. 1 for details). Due to the complexity and long-time frames with reconstruction procedures, this was done to simply identification of confounding factors that can impact results. An overall risk of bias rating of low, some concerns, high or very high risk was conferred for each study through accumulation of ratings for all domains as per the tool algorithm [34, 35]. Data was narratively synthesized. Meta-analysis could not be performed due to inconsistency in outcome measures and variables available.

### Instrument study selection and analysis

#### Content validity study selection

**Fig. 2:**
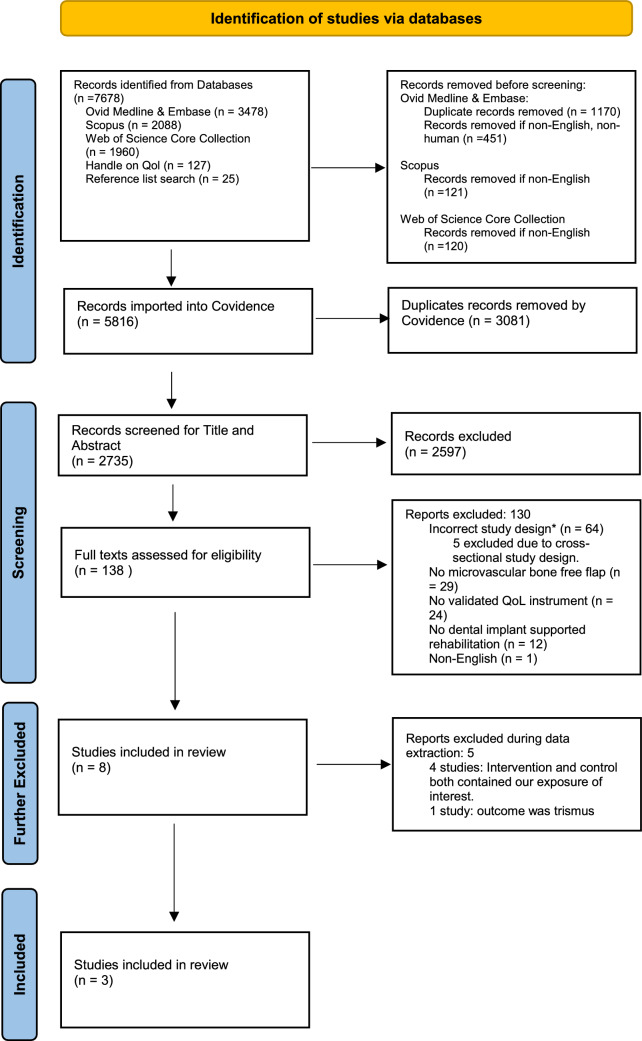
PRISMA 2020 flow diagram for new systematic reviews which included searches of databases and registers only. Adapted from the PRISMA 2020 Statement [68].*Case series, cross-sectional, non-intervention comparator (pre-treatment QoL/pre-surgery QoL), grouping of microvascular free flap and local flaps and grouping of implant-based rehabilitation with other rehabilitation types (removable denture, obturator)

PROM instruments from the three included studies and also those that appeared at least twice in the excluded cross-sectional studies and the 4 studies where intervention and control both contained our exposure of interest (Fig. 2) were analyzed to capture the most frequently used instruments on this topic. These were the EORTC QLQ 30 H&N 35, UW-QOL and OHIP-14. A search strategy was adapted from the systematic review search strategy (Appendix 1a) and combined with the COSMIN search filter to identify all available content validity studies on maxillofacial reconstruction and related populations such as conventional prosthesis or pathology-based studies in PubMed and Embase for these instruments (Appendix 1b) [36]. The search results were exported into Covidence and duplicates removed. Two authors (SA and YMK) independently screened for title and abstract and then full text to extract data from validation studies. SA and YMK also independently conducted the COSMIN analysis. Findings were discussed for consensus and conflicts were resolved in consultation with AS.

#### COSMIN analysis

Consensus based Standards for the selection of health Measurement Instruments (COSMIN) methodology was used to determine the content validity of existing PROM instruments for maxillofacial reconstruction [37–41]. Each instrument received an overall rating for general PROM instrument development as very good (important aspects well described in development studies), adequate (important aspects may not be described but can be assumed), doubtful (important aspects not clearly described), inadequate (important aspects missing) or not applicable. This was based on 1) PROM design (item generation) and 2) cognitive interview or pilot study. Instrument development, content validity studies and the PROM instrument itself were then examined against the following content validity criteria:

1. Construct of interest: QoL
2. Population of interest: maxillofacial reconstruction (defined as bone or soft tissue free flap reconstruction plus dental rehabilitation with implants following resection of malignant or benign pathology of the maxilla or mandible)
3. Context of interest: for use in research trials and as a QoL tool in clinical practice for patients receiving maxillofacial reconstruction

Instrument development studies, validation studies and the PROM instrument itself were rated ( +) if ≥ 85% of the items of the PROM (or subscale) fulfill the criterion, (-) if < 85% of the items of the PROM (or subscale) fulfill the criteria and (?) if not enough information available or quality of (part of a) the study inadequate, for the following categories:

1. Relevance = relevance to the construct, population and context of interest,
2. Comprehensiveness = are all key concepts included within the items/subscales,
3. Comprehensibility = how well the instrument is understood.

An overall rating of sufficient ( +), insufficient (-) or inconsistent (± = some sufficient and insufficient elements) was given for these categories. The quality of available evidence for relevance/comprehensiveness/comprehensibility ratings were graded as high, moderate, low or very low based on the available instrument development studies, content validity studies and reviewers rating of the instrument itself (lower level of evidence).

## Results

### Study selection

Figure 2 shows the PRISMA flowchart of the study screening and selection process. After removing duplicates, 2,735 articles title and abstract were screened and a total of 138 articles full text screened with 130 of these excluded. Reasons for exclusion were incorrect study design for 64 studies, intervention having no microvascular free flap (n = 29), no validated instrument (n = 24), no dental implant supported rehabilitation (n = 12) and not published in English (n = 1). A further four studies were excluded during data extraction, as both the intervention and control groups in these studies contained our exposure of interest [9, 42–44]. One study was excluded as the outcome was trismus (reduced mouth opening), not QoL [45]. The remaining three studies were included in the systematic review.

### Characteristics of the included studies

Of the three included studies, two were cohort studies and one RCT (Table 1). These studies were conducted in populations from middle-high income countries (Canada and Netherlands, China and Taiwan and Italy) [8, 46, 47]. Pathologies mostly located in the mandible were reported, with predominantly malignant indications. Radiation therapy and smoking status were inconsistently reported.

**Table 1:** Study Characteristics; *NR: Not reported; **ART: Alberta reconstruction technique

1st Author Publication Year	Study type	Country	Age (years)	Gender	Smoking	Sample Size	Pathology Type	Pathology(s) ICD classification	Defect type
De Groot 2020	Prospective Cohort	Intervention: Canada Control: Netherlands	Intervention mean age 45.0 ± 14.3 Control mean age 70.8 ± 13.6	Intervention: 3 Male 8 Female Control: 7 Male 6 Female	NR^*^	N = 24 (N = 11 Intervention: ART** = 6, Rohner = 5) (N = 13 control)	Malignant and Benign	Squamous cell carcinoma Non-SCC malignancy Benign lesions	Maxillectomy
Pappalardo 2018	Prospective Cohort	China and Taiwan	Intervention mean 32.3 ± 12.7 Control mean 34.7 ± 15.7	Intervention: 13 Male 8 Female Control: 9 Male 4 Female	NR	N = 34 (N = 22 Intervention) (N = 12 control)	Benign	Ameloblastoma	Mandibulectomy
Ciocca 2015	Noninferiority RCT	Italy	Intervention range 29—61 Control range 34–68	8 Male 2 Female	NR	N = 10 (N = 5 Intervention) (N = 5 control)	Malignant	Squamous cell carcinoma	Mandibulectomy

All three studies reported dental rehabilitation involving implants in microvascular fibula free flaps (MFFF) (Table 2). Implants were placed across immediate and delayed time frames following reconstruction surgery. The number of implants placed, and timing of prosthesis placement were also variable. The comparator group was tissue reconstruction only with no dental rehabilitation in Pappalardo et al. [8], removable denture following tissue reconstruction in Ciocca et al. [47] and obturator prosthesis in De Groot et al.[46]. The PROM instruments used were versions of the EORTC QLQ 30 H&N 35 in De Groot et al. [46] and Ciocca et al. [47] and UW-QOL and OHIP-14 in Pappalardo et al. [10]. De Groot et al. [46] reported PROM instrument administration during post-operative follow-up period of 914 ± 662 days for exposure and 365 ± 11 days for control groups. Pappalardo et al. [8] administered instruments during follow-up period of 100.2 ± 42.1 months for exposure and 68.8 ± 45.8 months for control groups. Ciocca et al. [47] did not specify this.

**Table 2:** Study Main Findings; ^*^MFFF: Microvascular fibula free flap; ^**^NA: Not Applicable

1st Author Publication Year	Exposure group	Implant		Prosthesis		Comparator	Follow-up Period	QoL Instrument	Radiation Therapy		Outcome Measure	Uncertainty Type	Variable	Outcome Measure Value	Uncertainty ( ±)	P value	Reported Limitations
		Number	Timing	Type	Timing of Loading				Yes/No	Timing (pre/post op)							
De Groot 2020	ART: phase 1 surgery involving MFFF^*^ with implant insertion and loading at 6 months during phase 2 Rohner: phase 1 surgery involving prefabricated fibula with implants insertion and time allowed for osseointegration and phase 2 involving MFFF harvest, reconstruction and loading at 4–6 weeks	NR	ART (during 1st phase of surgery) Rohner (during 1st phase of surgery)	Fixed and Removable	ART protocol (6 months after phase 1 surgery) Rohner protocol (4–6 weeks after phase 1)	Obturator	Exposure: mean days 914 ± 662 Control: mean days 365 ± 11	EORTC H & N 35	Yes for ART	NR	Mean QoL scores (low correlated with high QoL)	Standard deviation	Overall EORTC H & N 35 QoL Score Intervention Group	13.7	9.2	0.001	Low sample number Bias due to different surgical teams cultural differences Selection bias of dentally rehabilitated patients only
													Overall EORTC H & N 35 QoL Score Control Group	34.5	9.8		
Pappalardo 2018	Implant supported fixed prosthesis with MFFF	Total 65 across sample, 30 osseointegrated implants primarily and 35 placed secondarily	Immediate and delayed	Fixed	1 month following implant osseointegration	MFFF only	Total 89.1 ± 45.4 months Exposure: 100.2 ± 42.1 Control: 68.8 ± 45.8	UW-QOL, OHIP-14	No	NA**	Mean QoL scores (low correlated with high QoL) ± Standard Deviation	Standard deviation	Overall OHIP 14 QoL Score Intervention Group	NR	NR	< 0.05	In exposure group one patient underwent secondary orthognathic surgery to correct malocclusion due to following-up mandible growth and three patients underwent secondary flap debulking procedures to improve facial symmetry. Two patients underwent vertical distraction osteogenesis of the fibula graft to increase bone height for dental implant placement
													Overall OHIP 14 Qol Score Control Group	NR	NR		
													Overall UW-QOL Score Intervention Group	NR	NR	< 0.05	
													Overall UW-QOL Score Control Group	NR	NR		
Ciocca 2015	Implant supported fixed prosthesis with MFFF	8 to 4 per patient	NR	Fixed	NR	Removable prosthesis with MFFF	NR	EORTC H & N QLQ 30	No	NA	NR	NR	Overall QoL Score Intervention Group	NR	NR	< 0.05	Small sample size
													Overall QoL Score Control Group	NR	NR		

### Effect of maxillofacial reconstruction on QoL

**Fig. 3:**
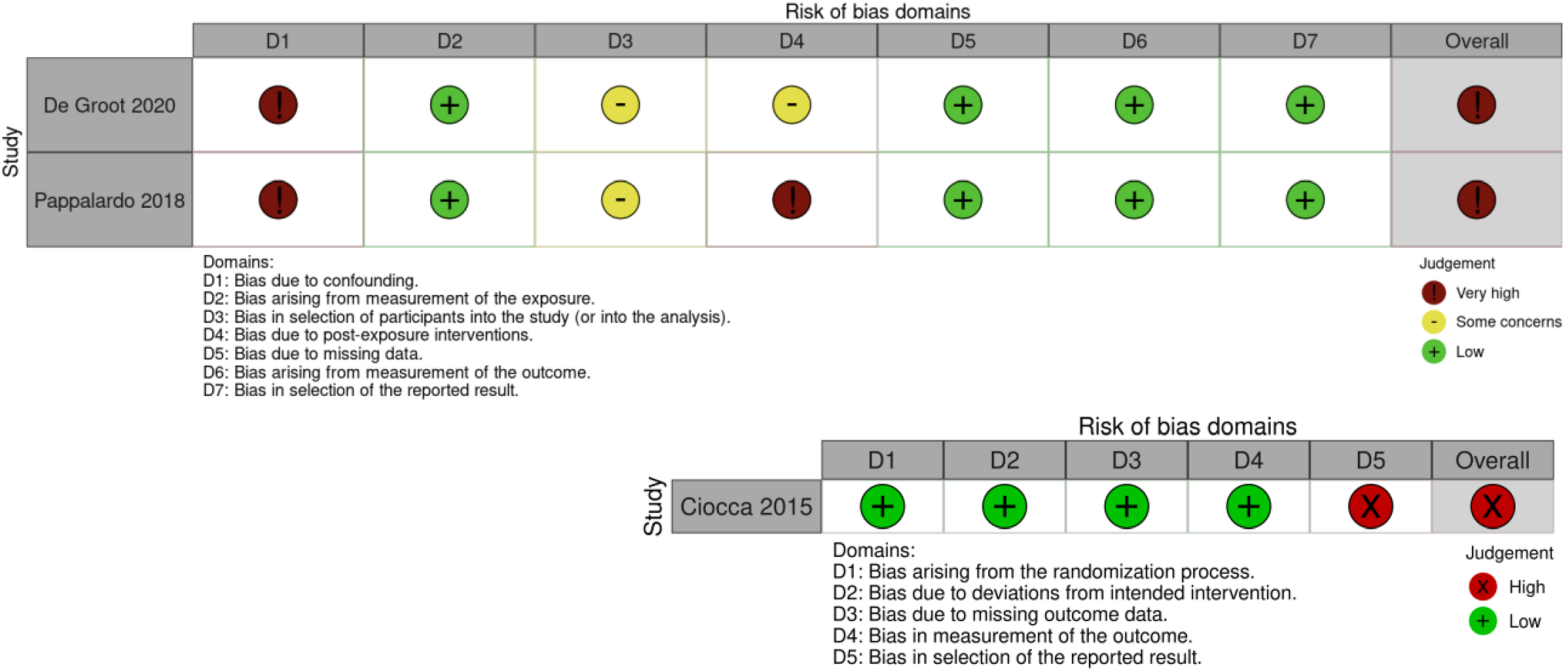
Study Quality Assessment Plots for the two included cohort studies (upper) and one RCT (lower)

The two cohort studies reported an association between maxillofacial reconstruction and improved HRQoL and OHRQoL. De Groot et al. [46] found overall improved mean EORTC QLQ H&N 35 QoL scores with maxillofacial reconstruction (13.7 ± 9.2) compared to obturator (34.5 ± 9.8), with lower score values indicating higher QoL. Improvement was also seen in the QoL domain on “sexuality” (3.0 ± 6.7 vs 44.8 ± 40.5), “feeding tube” (9.1 ± 30.2 vs 84.6 ± 37.6), “weight gain” (0 vs 84.6 ± 37.6) and “weight loss” (18.2 ± 40.5 vs 69.2 ± 48.0) in reconstruction compared to obturator group. However, the study was assessed as having overall very high risk of bias due to confounding (Fig. 3).

Pappalardo et al. [8] reported QoL improvement in the reconstruction compared to no dental rehabilitation group on OHIP-14 (lower score values indicated higher QoL) in the domain of “physical disability” (22.2 ± 21.7 vs 38.5 ± 23.3, P < 0.01), “psychological discomfort” (26.1 ± 21.5 vs 37.5 ± 23.3, P = 0.02) and “physical pain” (22.6 ± 18.2 vs 31.3 ± 24.7, P = 0.05). “Chewing” (86.4 ± 22.8 vs 62.5 ± 29.2, p = 0.01), “activity” (88.6 ± 12.7 vs 77.1 ± 19.8, P = 0.02), and “anxiety” (87.7 ± 15.1 vs 72.5 ± 27.7, P = 0.02) were better in the control. Overall mean OHIP-14 and UW-QOL scores for intervention and control groups were not reported. The study obtained a very high risk of bias ROBINS-E rating due to unadjusted confounding and post-exposure interventions (other corrective surgeries conducted in the exposure group such as orthognathic, debulking and distraction surgeries).

Ciocca et al. [47] found no difference in the mean EORTC H&N30 scores between the reconstruction and removable denture groups. The study received an overall high risk of bias RoB 2 rating due to bias in selection of reported results. This was because the numerical values corresponding to QoL assessment comparing intervention and control groups were not reported. The authors were contacted for clarification, but no response was received. A common limitation in all included studies was small sample size (Table 2).

## Instrument analysis

### Validation studies

For the three PROM instruments (EORTC QLQ 30 H&N 35, UW-QOL and OHIP-14), 845 search results were exported into Covidence and 107 duplicates removed. Title and abstract (n = 738) and then full text (n = 28) screening resulted in data extraction from 21 validation studies. There are no published validation studies for EORTC QLQ 30 H&N 35, UW-QOL and OHIP-14 on maxillofacial reconstruction. The extracted studies on related populations are presented in Table 3.

**Table 3:** COSMIN Analysis; *Content validity was assessed for construct of interest = QoL, population of interest = surgical maxillofacial reconstruction (bone/soft tissue free flap reconstruction plus dental rehabilitation) and context of interest = for use in research trials and as a QoL tool in clinical practice in patients receiving maxillofacial reconstruction

	PROM Instrument Characteristics						Validation studies	PROM Instrument COSMIN Analysis				
PROM	Original Construct	Original Target population	Original Context of use	Number of items and Subscales	Recall period	Response options	Validated Population	General PROM Development Rating	Content Validity^*^			Quality of Evidence for Relevance, Comprehensiveness and Comprehensibility
									Relevance Rating	Comprehensiveness Rating	Comprehensibility Rating	
EORTC H & N 35 + EORTC QLQ 30[48, 49, 70]	HRQoL	Head and neck cancers (larynx, oral, pharynx, sinus, salivary glands)	For use in clinical trials before, during, and after in patients receiving combinations of radiotherapy, surgery, or chemotherapy	EORTC H& N 35: 35 items Subscale: · Pain · Swallowing · Senses · Saliva · Trismus · Speech · Social eating · Social contact · Sexuality · Nutrition EORTC QLQ30: 30 items Subcale: · Physical functioning · Role functioning · Cognitive functioning · Emotional functioning · Social functioning · Fatigue · Pain · Nausea and Vomiting · Global QOL	Past week	EORTC H&N 35: 4-point Likert-like and yes/no EORTC QLQ30: 4 point Likert	No content validity studies on maxillofacial reocnstruction Related content validity studies: · Carrillo 2013: Spanish translation, various HN cancers grouped, awaiting treatment[71] · Chie 2003: Taiwanese version, nasopharyngeal carcinoma, undergoing radio/chemotherapy[72] · Jensen 2006: Danish version, various HN cancers grouped, unspecified surgery[73] · Zahid 2022: Urdu version, various HN cancers grouped, unspecified surgery, undergoing radio/chemotherapy[74]	**Adequate** Head and neck oncology patient and expert involvement in concept elicitation, comprehensiveness and comprehensibility analysis	**Overall: ± Inconsistent** PROM development studies: ± Sufficient evidence for QoL however insufficient evidence for maxillofacial reconstruction as originally designed for head and neck oncology population. Insufficient evidence for context of use as intended for research applications rather than clinical Validation studies: None available for maxillofacial reconstruction Reviewers rating of PROM instrument: Some relevant items, others may be unnecessary. Unable to determine if > 85% of items are relevant for maxillofacial reconstruction	**Overall: + sufficient** PROM development studies: Head and neck oncology patient and experts involved in instrument comprehensiveness analysis Validation studies: None available for maxillofacial reconstruction Reviewers ratings of PROM instrument: comprehensive concept coverage, most of which likely apply to maxillofacial reconstruction, however cannot definitively conclude if all key maxillofacial reconstruction concepts are included	**Overall: + Sufficient** PROM development studies: Head and neck oncology patients involved in item comprehensibility analysis Validation studies: None available for maxillofacial reconstruction Reviewer ratings of PROM instrument: > 85% of the items appear to be appropriately worded	**Moderate** Evidence mainly from PROM development studies and reviewer ratings of instrument itself, however no content validity studies available for maxillofacial reconstruction
EORTC H & N 43 (latest version of EORTC H&N 35) + EORTC QLQ 30 (same as above)	Same as above	Same as above	Same as above	43 items Subscales: same as above EORTC QLQ30: 30 items Subscales: same as above	Same as above	Same as above	No content validity studies on maxillofacial reconstruction Related content validity studies: Davudov 2020: · Azerbaijan translation, oral cancer patients, resection + unspecified flap reconstruction [75, 76] · Zahid 2022: Urdu version, various HN cancers grouped, unspecified surgery, undergoing radio/chemotherapy [74]	**Adequate** Head and neck oncology patient and expert involvement in concept elicitation, comprehensiveness and comprehensibility analysis	**Overall: ± Inconsistent** PROM development studies: Sufficient evidence for QoL however insufficient evidence for maxillofacial reconstruction as originally designed for head and neck oncology population. Insufficient evidence for context of use as intended for research applications rather than clinical Validation studies: None available for maxillofacial reconstruction Reviewers rating of PROM instrument: Some relevant items, others may be unnecessary. Unable to determine if > 85% of items are relevant for maxillofacial reconstruction	**Overall: + sufficient** PROM development studies: Head and neck oncology patient and experts involved in concept and item generation Validation studies: None available for maxillofacial reconstruction Reviewers ratings of PROM instrument: comprehensive concept coverage, most of which likely apply to maxillofacial reconstruction, however cannot definitively conclude if all key maxillofacial reconstruction concepts are included	**Overall: + Sufficient** PROM development studies: Head and neck oncology patients involved in item comprehensibility analysis Validation studies: None available for maxillofacial reconstruction Reviewer ratings of PROM instrument: > 85% of the items appear to be appropriately worded	**Moderate** Evidence mainly from PROM development studies and reviewer ratings of instrument itself, however no content validity studies available for maxillofacial reconstruction
University of Washington Quality of Life Questionnaire -version 4 (UW QOL v4)[77, 78]	HRQoL	Head and neck cancer patients in all stages and with varied tumor sites	For use in broad group of head and neck cancer patients to provide a standardised basis for comparison of treatment outcomes across tumour sites and stages	17 items: Subscales: · Pain · Appearance · Activity · Recreation · Swallowing · Chewing · Speech · Shoulder · Taste · Saliva · Mood · Anxiety · Global QOL items · Other issues in free text	Past week	Range from 0 for worst to 100 for best and written responses	No content validity studies on maxillofacial reoconstruction Related content validity studies: · Lee 2017: 36 of 211 patients had tumour resection, lymph node dissection and reconstruction with free flap, others had resection only ± lymph node dissection ± radio/chemotherapy[79] · Adnane 2016: Moroccan translation, unspecified surgery ± radio/chemotherapy, various HN cancers grouped[80] · Katre 2008: Appearance domain, oropharyngeal carcinoma, unspecified surgery[81] · Rogers 1998: 25 of 29 had unspecified free flap following resection of oral cancer ± adjuvant radiotherapy[82] · Rogers 2010: Physical and social-emotional subscale validation, 291 of 838 patients had unspecified free flap surgery for oral/oropharyngeal cancers ± radio/chemotherapy[83] · Thomas 2009: Speech domain, various HN cancers grouped, unspecified free flap in 48 of 77 patients[84] · Thomas 2008: swallow domain, various HN cancers, unspecified free flap in 48 of 77 patients[85] · Zuydam 2012: Swallow domain, oral and oropharyngeal carcinoma, 68 of 94 patients had unspecified free flap surgery[86] · D’cruz 2007: Marathi and Hindi versions, various HN cancer patients[87] · Nazar 2010: Spanish translation, various HN cancers, unspecified surgery, radiation, chemotherapy[88] · Senkal 2012: Turkish translation, various HN cancers, unspecified surgery, radiation, chemotherapy[89] · Vartanian 2006: Brazilian–Portuguese translation, various HN cancers, unspecified surgery, radiation, chemotherapy [90]	**Doubtful** Patient or expert involvement in concept elicitation, comprehensiveness and patient comprehensibility analysis of items not specified during PROM development	**Overall: ± Inconsistent** PROM development studies: Sufficient evidence for QoL and possibly for context of use however insufficient evidence for maxillofacial reconstruction as originally designed for head and neck oncology population Validation studies: None available for maxillofacial reconstruction Reviewers rating of PROM instrument: > 85% of items appear relevant for maxillofacial reconstruction	**Overall: ± inconsistent** PROM development studies: patient or expert involvement in concept generation for comprehensiveness analysis not specified Validation studies: None available for maxillofacial reconstruction Reviewers rating of PROM instrument: Although items appear to cover some concept subscales for maxillofacial reconstruction, instrument lacks potentially key subscales such as nutrition, social impact etc	**Overall: ± Inconsistent** PROM development studies patient involvement in comprehensibility analysis of items not specified Validation studies: None available for maxillofacial reconstruction Reviewers rating of PROM instrument: > 85% of the items appear to be appropriately worded	**Low** Evidence mainly from reviewer ratings of instrument itself, as no content validity studies available for maxillofacial reconstruction and PROM development studies being of doubtful quality
Oral Health Impact Profile – 14 (OHIP 14)[91]	OHRQoL	Dentate and edentulous patients	Designed to measure patient's perception of the social impact of oral disorders on their well-being	14 items Subscales: · Functional limitation · Physical pain · Psychological discomfort · Physical disability · Psychological disability · Social disability · Handicap	Past year	5-point Likert	No content validity studies on maxillofacial reconstruction Related content validity studies: · Tesic 2020: Various HN cancers grouped, unspecified surgery ± radio/chemotherapy[92] · Edentulous patients, dentures and implant supported prosthesis grouped [93]	**Doubtful** Patient involvement in item reduction. Otherwise, patient or expert involvement in concept elicitation, comprehensiveness and patient comprehensibility analysis of items not specified during PROM development	**Overall: ± Inconsistent** PROM development studies: Sufficient evidence for QoL however insufficient evidence for maxillofacial reconstruction as originally designed for target population of dentate and edentulous individuals Validation studies: None available for maxillofacial reconstruction Reviewers rating of PROM instrument: > 85% of items appear relevant for maxillofacial reconstruction	**Overall: ± Inconsistent** PROM development studies: patient or expert involvement in concept or item generation not specified Validation studies: None available for maxillofacial reconstruction Reviewers rating of PROM instrument: Although items appear to cover some concept subscales for maxillofacial reconstruction, instrument lacks potentially key subscales such as speech, swallowing etc	**Overall: ± Inconsistent** PROM development studies: patient involvement in comprehensibility analysis of items not specified Validation studies: None available for maxillofacial reconstruction Reviewers rating of PROM instrument: > 85% of the items appear to be appropriately worded	**Low** Evidence mainly from reviewer ratings of instrument itself, as no content validity studies available for maxillofacial reconstruction and PROM development studies being of doubtful quality

### COSMIN analysis

#### PROM development

EORTC QLQ 30 H&N 35 (and version EORTC H&N 43) has ‘adequate’ development with patient and expert involvement in concept elicitation, comprehensiveness and comprehensibility analysis. However, concept elicitation involved structured patient interviews, whereas COSMIN recommends qualitative interview methodology for item generation with detailed use of moderator, topic guide, transcription and coding [40, 48]. UW-QOL and OHIP-14 were rated as ‘doubtful’ as key elements regarding patient and expert involvement in PROM development were not specified in development studies (Table 3).

#### Content Validity Analysis for maxillofacial reconstruction

Relevance Rating: All three instruments were rated ‘inconsistent’ for relevance. All three capture QoL, with EORTC QLQ 30 H&N 35 and UW-QOL assessing HRQoL and OHIP-14 assessing OHRQoL. However, they were originally designed for other target populations (EORTC QLQ 30 H&N 35 and UW-QOL for head and neck oncology and OHIP-14 for dentate/edentulous patients) and have not been validated in patient populations that have undergone maxillofacial reconstruction.

Comprehensiveness Rating: Two instruments (UW-QOL and OHIP-14) were rated ‘inconsistent’ as they contain some but lack potentially other key concepts (e.g. nutrition, social impact, speech, swallowing), with no patient or expert involvement in item/subscale generation specified in instrument development studies. EORTC QLQ 30 H&N 35 was rated ‘sufficient’ as it contains concept subscales generated through head and neck oncology patient and expert involvement, most of which are likely applicable to maxillofacial reconstruction based on reviewers rating of the PROM instrument. However, none of these PROM instruments have had maxillofacial reconstruction patient involvement in comprehensiveness analysis, hence it cannot be definitively concluded that all key maxillofacial reconstruction QoL concepts are covered in these instruments.

Comprehensibility Rating: EORTC QLQ 30 H&N 35 was rated ‘sufficient’ as it has been tested on head and neck oncology patients for comprehensibility and the reviewers rated > 85% of the items as appropriately worded. UW-QOL and OHIP-14 received an ‘inconsistent’ rating since no comprehensibility analysis involving any population is specified in the development studies, however the reviewers rated > 85% of the items as appropriately worded.

#### Evidence

The quality of evidence for this COSMIN analysis was graded as ‘moderate’ for EORTC QLQ 30 H&N 35 as it came from instrument development studies and the reviewers rating of the instrument itself since no content validity studies on maxillofacial reconstruction are available. For UW-QOL and OHIP-14, it was graded as ‘low’ as it came mainly from the reviewers rating of the instrument, with no content validity studies and instrument development studies being of doubtful quality.

## Discussion

Our systematic review found that although existing cohort studies show maxillofacial reconstruction is associated with improved QoL compared to obturator prosthesis and no dental rehabilitation, these studies have high risk of bias due to uncontrolled confounding. The studies also have heterogenous methodology including the QoL instrument utilized to capture outcome data. Hence, the quality of the studies is an issue which prevents conclusive findings from being drawn from the existing literature. The COSMIN analysis has shown that EORTC QLQ 30 H&N 35, UW-QOL and OHIP-14, currently used to capture data in this population, have not been validated for use in maxillofacial reconstruction. Hence, the conclusiveness of the findings also needs to be interpreted with this in mind.

Our findings provide new insights into this topic. A systematic review by Wijbenja et al. [16] found the retrospective nature of included studies was a concern while reporting good to excellent speech intelligibility and aesthetics with reconstruction compared to conventional denture. Another systematic review by Quadri et al. [17] found no substantial evidence on the OHRQoL impacts of dental prosthetic rehabilitation in cancer patients as adequate control group analysis was missing in many of their included studies. A review by Said et al. [15] found no improvement in general QoL with dental implant rehabilitation compared to pre-operative status, acknowledging their analysis was limited by inclusion of studies capturing QoL data with and without validated instruments, which created variability in measurement of outcomes. None of these reviews performed an analysis on the measurement properties of QoL instruments utilised in existing studies. Our review has progressed knowledge in this area by highlighting the reasons why it is difficult to synthesise evidence on this topic, including lack of confounding analysis and heterogenous instrument utilisation in the available studies, with existing QoL instruments not being validated in maxillofacial reconstruction.

Our systematic review employed a rigorous methodology. The authors developed a broad search strategy and conducted comprehensive searches across multiple databases to ensure that published literature on this topic was not missed. This resulted in a large number of published studies being screened. Study quality assessment was also thoroughly performed with the aid of a DAG. Adjustment for confounding factors, especially pre-implantation radiation therapy and smoking is necessary as these are risk factors for dental implant complications which can adversely impact patient QoL [49, 50]. Other factors including age, gender, co-morbidities, disease prognosis and financial status should also be considered [51–55]. Without adjustment for confounding, the true impact of the intervention cannot be determined as it may be over or under estimated.

Another strength of this study is the COSMIN content validity analysis, which to the best of our knowledge, is the first ever published on this topic. EORTC QLQ 30 H&N 35 satisfied more COSMIN criteria than UW-QOL and OHIP-14. However, none of these instruments have sufficient relevance for maxillofacial reconstruction, having been designed for other target populations. There are no published content validity studies for these instruments on this population. The existing instruments, especially EORTC H&N 35 QLQ 30, have a role as they are currently the only means of gathering QoL data in maxillofacial reconstruction. Validating existing instruments would provide evidence towards their appropriateness for this population. However, the incorporation of dental rehabilitation in maxillofacial reconstruction may contribute to an interface for new QoL considerations and dynamic changes depending on the treatment and recovery stage. Existing generic instruments may not detect these differences and non-generic instruments may be too narrowly focused to enable comparisons across interventions. A tailored PROM instrument may be able to capture patient perceptions of QoL impacts within this population group, especially when comparing different interventions.

This review included only three studies, and this was partly due to the exclusion of cross-sectional studies and studies capturing QoL without validated instruments. Studies capturing QoL through author-made questionnaires and verbal interviews were excluded, as this would compound standardization of outcomes in our review. There are numerous cross-sectional studies that show the positive QoL impacts of maxillofacial reconstruction involving dental implant rehabilitation on mastication, swallowing and speech, facial and dental aesthetics and reduced psycho-social issues associated with deformity and loss of teeth [9, 11–13, 41, 44, 57–62]. However, causal relationships over time cannot be detected through these studies since the exposure and outcome are assessed at a single time point and therefore cross-sectional studies were excluded from this systematic review. Estimating causal effects of oral rehabilitation with dental implants on QoL is necessary to conclude that a positive change in QoL for patients is achieved due to this intervention. QoL changes are dynamic throughout the post-surgery period as patients learn to adapt to functioning again and so the impact of dental rehabilitation is unlikely to be consistent. The QoL impact will also wary depending on the procedures undertaken and timing of instrument administration. Causally focused observational cohort studies or randomized trials can help quantify such causal effects.

The authors decision to include English language papers only may contribute to some form of publication selection bias, however the vast majority of international literature is in English and therefore the extent of such a bias should be small. Another limitation of this paper is that the type of statistical analysis performed by the included studies was not assessed. Parametric statistics may be used to analyse data that is not normally distributed as with QoL measurements. An in-depth assessment of this was not among the objectives of this study. Our review only considered tools applied within studies of any epidemiological design found within our search strategy.

Our paper has highlighted some key research implications. Existing PROM instruments should be first validated in maxillofacial reconstruction prior to their use in future relevant studies. Ideally, a tailored PROM instrument should be designed to capture QoL data across different interventions in maxillofacial reconstruction, which can better support clinical decision-making processes based on best treatment outcomes. High quality cohort studies should be conducted to determine the longer-term impact of these complex interventions on people’s QoL and potentially inform intervention selection.

## Conclusion

Cohort studies show an association between maxillofacial reconstruction involving dental implant rehabilitation and improved QoL. However, due to a high risk of bias, the impact cannot be concluded definitively and further research in this area is needed. Existing PROM instruments lack content validity for use in maxillofacial reconstruction, highlighting the need for tailored PROM instruments for QoL evaluation in maxillofacial reconstruction.

## Supplementary Information

Below is the link to the electronic supplementary material.

Supplementary file1 (DOCX 76 kb)

## Data Availability

The authors confirm that the data supporting the findings of this study are available within the article [and/or] its supplementary materials.
