# Supplementary material for: Impact of rehabilitation with dental implants on the quality of life of patients undergoing maxillofacial reconstruction: a systematic review
Source: Qual Life Res. 2024 Oct 17;34(1):113–30. doi: 10.1007/s11136-024-03795-w (PMC11802631; doi:10.1007/s11136-024-03795-w)
Supplement: Supplementary file 1 — Supplementary file1 (DOCX 76 kb) [file 11136_2024_3795_MOESM1_ESM.docx]

**Appendix 1**

**Appendix 1a: Systematic Review Search Strategy**

|  | **Concept 1** | **Concept 2** |
| --- | --- | --- |
| **Synonyms/keywords** | Dental impl*  Implant supported*  Implant retained*  Dental prosthe* Fixed prosthe*  Dental rehabilitation  Mouth rehabilitation  Oral rehabilitation  Mandible prosthe*  Prosthetic rehabilitation  Implant borne*  Implant denture*  Fixed denture*  Implant overdenture* | QOL  Quality of life  HRQOL  OHRQOL  Oral health related quality of life  Health related quality of life  Validated tool*  Patient reported outcome measure*  Patient reported outcome*  PROMs  Self reported outcom*  Oral Health Impact Profile*  University of Washington  European Organisation for Research and Treatment of Cancer  UWQ*  OHIP*  EORTC*  VAS scal*  EuroQol  WHOQOL |
| MeSH terms | Dental Prosthesis, Implant-Supported  Dental Implants  Mandibular prosthesis  Mouth Rehabilitation | Quality of Life  Patient Reported Outcome Measures |
| Emtree | Full mouth rehabilitation  Tooth implant  Endosseous implant  Implant supported denture | Quality of life  Exp Quality of life assessment  Patient reported outcome |

**Databases**

**Embase Classic+Embase (1947 to January 05 2024) Ovid MEDLINE(R) ALL (1946 to January 05, 2024)**

| **#** | **Searches** | **Results** |
| --- | --- | --- |
| 1 | Quality of life/ | 922791 |
| 2 | Patient Reported Outcome Measures/ | 64661 |
| 3 | Dental Prosthesis, Implant-Supported/ | 12411 |
| 4 | Dental Implants/ | 42315 |
| 5 | Mandibular Prosthesis/ | 2030 |
| 6 | Mouth Rehabilitation/ | 1889 |
| 7 | Dental implant*.mp. | 73801 |
| 8 | Implant supported*.mp. | 20511 |
| 9 | Implant retained*.mp. | 2743 |
| 10 | Dental rehabilitation.mp. | 1662 |
| 11 | Mouth rehabilitation.mp. | 2447 |
| 12 | Oral rehabilitation.mp. | 3679 |
| 13 | implant-supported denture/ | 12530 |
| 14 | full mouth rehabilitation/ | 438 |
| 15 | Implant borne*.mp. | 402 |
| 16 | Implant denture*.mp. | 308 |
| 17 | QOL.mp. | 157001 |
| 18 | Quality of life.mp. | 1292142 |
| 19 | HRQOL.mp. | 62510 |
| 20 | OHRQOL.mp. | 4504 |
| 21 | Oral health related quality of life.mp. | 7551 |
| 22 | Health related quality of life.mp. | 151641 |
| 23 | Validated tool*.mp. | 11513 |
| 24 | Patient reported outcome measure*.mp. | 42263 |
| 25 | Patient reported outcome*.mp. | 123427 |
| 26 | PROMs.mp. | 13510 |
| 27 | Self reported outcom*.mp. | 3130 |
| 28 | "quality of life"/ or exp "quality of life assessment"/ | 977454 |
| 29 | patient-reported outcome/ | 71674 |
| 30 | Oral Health Impact Profile*.mp. | 4355 |
| 31 | OHIP*.mp. | 4836 |
| 32 | University of Washington*.mp. | 6949 |
| 33 | UWQ*.mp. | 183 |
| 34 | "European Organisation for Research and Treatment of Cancer".mp. | 4884 |
| 35 | EORTC*.mp. | 34396 |
| 36 | VAS scal*.mp. | 4317 |
| 37 | EuroQol.mp. | 21148 |
| 38 | WHOQOL.mp. | 11037 |
| 39 | tooth implant/ | 20423 |
| 40 | endosseous implant/ | 18958 |
| 41 | mandibular prosthe*.mp. | 1454 |
| 42 | Fixed denture*.mp. | 929 |
| 43 | Implant overdenture*.mp. | 1364 |
| 44 | 3 or 4 or 5 or 6 or 7 or 8 or 9 or 10 or 11 or 12 or 13 or 14 or 15 or 16 or 39 or 40 or 41 or 42 or 43 | 97223 |
| 45 | 1 or 2 or 17 or 18 or 19 or 20 or 21 or 22 or 23 or 24 or 25 or 26 or 27 or 28 or 29 or 30 or 31 or 32 or 33 or 34 or 35 or 36 or 37 or 38 | 1440517 |
| 46 | 44 and 45 | 3478 |
| 47 | limit 46 to english language | 3352 |
| 48 | limit 47 to human | 3027 |
| 49 | remove duplicates from 48 | 1857 |

**Scopus (updated 5/1/2024)**

| #41 | ( ( ( TITLE-ABS-KEY ( "Implant denture*" ) ) OR ( TITLE-ABS-KEY ( "Oral rehabilitation" ) ) OR ( TITLE-ABS-KEY ( "Mouth rehabilitation" ) ) OR ( TITLE-ABS-KEY ( "Dental rehabilitation" ) ) OR ( TITLE-ABS-KEY ( "Implant overdenture*" ) ) OR ( TITLE-ABS-KEY ( "implant borne*" ) ) OR ( TITLE-ABS-KEY ( "implant retained*" ) ) OR ( TITLE-ABS-KEY ( "Implant supported*" ) ) OR ( TITLE-ABS-KEY ( "Dental implant*" ) ) ) OR ( TITLE-ABS-KEY ( "Dental Prosthesis, Implant-Supported" ) ) OR ( TITLE-ABS-KEY ( "full mouth rehabilitation" ) ) OR ( TITLE-ABS-KEY ( "tooth implant" ) ) OR ( TITLE-ABS-KEY ( "endosseous implant" ) ) OR ( TITLE-ABS-KEY ( "mandibular prosthe*" ) ) OR ( TITLE-ABS-KEY ( "Fixed denture*" ) ) ) AND ( ( ( TITLE-ABS-KEY ( uwq* ) ) OR ( TITLE-ABS-KEY ( "University of Washington*" ) ) OR ( TITLE-ABS-KEY ( ohip* ) ) OR ( TITLE-ABS-KEY ( "Oral Health Impact Profile*" ) ) OR ( TITLE-ABS-KEY ( "Self reported outcom*" ) ) OR ( TITLE-ABS-KEY ( "Patient reported outcome*" ) ) OR ( TITLE-ABS-KEY ( "Patient reported outcome measure*" ) ) OR ( TITLE-ABS-KEY ( "Functional outcome*" ) ) OR ( TITLE-ABS-KEY ( "Validated tool*" ) ) OR ( TITLE-ABS-KEY ( "Health related quality of life" ) ) OR ( TITLE-ABS-KEY ( "Oral health related quality of life" ) ) OR ( TITLE-ABS-KEY ( ohrqol ) ) OR ( TITLE-ABS-KEY ( hrqol ) ) OR ( TITLE-ABS-KEY ( "Quality of life" ) ) OR ( TITLE-ABS-KEY ( qol ) ) OR ( TITLE-ABS-KEY ( whoqol ) ) OR ( TITLE-ABS-KEY ( euroqol ) ) OR ( TITLE-ABS-KEY ( "VAS scal*" ) ) OR ( TITLE-ABS-KEY ( eortc* ) ) OR ( TITLE-ABS-KEY ( proms ) ) ) OR ( TITLE-ABS-KEY ( "European Organisation for Research and Treatment of Cancer" ) ) OR ( TITLE-ABS-KEY ( "quality of life assessment" ) ) ) AND ( LIMIT-TO ( LANGUAGE , "English" ) ) | 1772 |
| --- | --- | --- |
| #40 | ( ( ( TITLE-ABS-KEY ( "Implant denture*" ) ) OR ( TITLE-ABS-KEY ( "Oral rehabilitation" ) ) OR ( TITLE-ABS-KEY ( "Mouth rehabilitation" ) ) OR ( TITLE-ABS-KEY ( "Dental rehabilitation" ) ) OR ( TITLE-ABS-KEY ( "Fixed denture*" ) ) OR ( TITLE-ABS-KEY ( "Implant overdenture*" ) ) OR ( TITLE-ABS-KEY ( "implant borne*" ) ) OR ( TITLE-ABS-KEY ( "implant retained*" ) ) OR ( TITLE-ABS-KEY ( "Implant supported*" ) ) OR ( TITLE-ABS-KEY ( "Dental implant*" ) ) ) OR ( TITLE-ABS-KEY ( "Dental Prosthesis, Implant-Supported" ) ) OR ( TITLE-ABS-KEY ( "full mouth rehabilitation" ) ) OR ( TITLE-ABS-KEY ( "tooth implant" ) ) OR ( TITLE-ABS-KEY ( "endosseous implant" ) ) OR ( TITLE-ABS-KEY ( "mandibular prosthe*" ) ) OR ( TITLE-ABS-KEY ( "Fixed denture*" ) ) ) AND ( ( ( TITLE-ABS-KEY ( uwq* ) ) OR ( TITLE-ABS-KEY ( "University of Washington*" ) ) OR ( TITLE-ABS-KEY ( ohip* ) ) OR ( TITLE-ABS-KEY ( "Oral Health Impact Profile*" ) ) OR ( TITLE-ABS-KEY ( "Self reported outcom*" ) ) OR ( TITLE-ABS-KEY ( "Patient reported outcome*" ) ) OR ( TITLE-ABS-KEY ( "Patient reported outcome measure*" ) ) OR ( TITLE-ABS-KEY ( "Functional outcome*" ) ) OR ( TITLE-ABS-KEY ( "Validated tool*" ) ) OR ( TITLE-ABS-KEY ( "Health related quality of life" ) ) OR ( TITLE-ABS-KEY ( "Oral health related quality of life" ) ) OR ( TITLE-ABS-KEY ( ohrqol ) ) OR ( TITLE-ABS-KEY ( hrqol ) ) OR ( TITLE-ABS-KEY ( "Quality of life*" ) ) OR ( TITLE-ABS-KEY ( qol ) ) OR ( TITLE-ABS-KEY ( whoqol ) ) OR ( TITLE-ABS-KEY ( euroqol ) ) OR ( TITLE-ABS-KEY ( "VAS scal*" ) ) OR ( TITLE-ABS-KEY ( eortc* ) ) OR ( TITLE-ABS-KEY ( proms ) ) ) OR ( TITLE-ABS-KEY ( "European Organisation for Research and Treatment of Cancer" ) ) OR ( TITLE-ABS-KEY ( "quality of life assessment" ) ) ) | 1926 |
| #39 | ( ( TITLE-ABS-KEY ( uwq* ) ) OR ( TITLE-ABS-KEY ( "university of washington*" ) ) OR ( TITLE-ABS-KEY ( ohip* ) ) OR ( TITLE-ABS-KEY ( "oral health impact profile*" ) ) OR ( TITLE-ABS-KEY ( "self reported outcom*" ) ) OR ( TITLE-ABS-KEY ( "patient reported outcome*" ) ) OR ( TITLE-ABS-KEY ( "patient reported outcome measure*" ) ) OR ( TITLE-ABS-KEY ( "functional outcome*" ) ) OR ( TITLE-ABS-KEY ( "validated tool*" ) ) OR ( TITLE-ABS-KEY ( "health related quality of life" ) ) OR ( TITLE-ABS-KEY ( "oral health related quality of life" ) ) OR ( TITLE-ABS-KEY ( ohrqol ) ) OR ( TITLE-ABS-KEY ( hrqol ) ) OR ( TITLE-ABS-KEY ( "quality of life*" ) ) OR ( TITLE-ABS-KEY ( qol ) ) OR ( TITLE-ABS-KEY ( whoqol ) ) OR ( TITLE-ABS-KEY ( euroqol ) ) OR ( TITLE-ABS-KEY ( "vas scal*" ) ) OR ( TITLE-ABS-KEY ( eortc* ) ) OR ( TITLE-ABS-KEY ( proms ) ) ) OR ( TITLE-ABS-KEY ( "european organisation for research and treatment of cancer" ) ) OR ( TITLE-ABS-KEY ( "quality of life assessment" ) ) | 757869 |
| #38 | ( ( TITLE-ABS-KEY ( "implant denture*" ) ) OR ( TITLE-ABS-KEY ( "oral rehabilitation" ) ) OR ( TITLE-ABS-KEY ( "mouth rehabilitation" ) ) OR ( TITLE-ABS-KEY ( "dental rehabilitation" ) ) OR ( TITLE-ABS-KEY ( "fixed denture*" ) ) OR ( TITLE-ABS-KEY ( "implant overdenture*" ) ) OR ( TITLE-ABS-KEY ( "implant borne*" ) ) OR ( TITLE-ABS-KEY ( "implant retained*" ) ) OR ( TITLE-ABS-KEY ( "implant supported*" ) ) OR ( TITLE-ABS-KEY ( "dental implant*" ) ) ) OR ( TITLE-ABS-KEY ( "dental prosthesis, implant-supported" ) ) OR ( TITLE-ABS-KEY ( "full mouth rehabilitation" ) ) OR ( TITLE-ABS-KEY ( "tooth implant" ) ) OR ( TITLE-ABS-KEY ( "endosseous implant" ) ) OR ( TITLE-ABS-KEY ( "mandibular prosthe*" ) ) | 60599 |
| #37  #36 | TITLE-ABS-KEY ( "Functional outcome*" ) TITLE-ABS-KEY ( whoqol ) | 60910  5,467 |
| #35 | TITLE-ABS-KEY ( euroqol ) | 8,298 |
| #34 | TITLE-ABS-KEY ( "VAS scal*" ) | 1,512 |
| #33 | TITLE-ABS-KEY ( eortc ) | 11,095 |
| #32 | TITLE-ABS-KEY ( uwq* ) | 85 |
| #31 | TITLE-ABS-KEY ( "University of Washington*" ) | 7,389 |
| #30 | TITLE-ABS-KEY ( ohip*) | 2,320 |
| #29 | TITLE-ABS-KEY ( "Oral Health Impact Profile*" ) | 2,122 |
| #28 | TITLE-ABS-KEY ( "Self reported outcom*" ) | 1,276 |
| #27 | TITLE-ABS-KEY ( "Patient reported outcome*" ) | 48,584 |
| #26 | TITLE-ABS-KEY ( "Patient reported outcome measure*" ) | 21,858 |
| #25 | TITLE-ABS-KEY ( "Validated tool*" ) | 4,186 |
| #24 | TITLE-ABS-KEY ( PROMs) | 11,658 |
| #23 | TITLE-ABS-KEY ( "Health related quality of life" ) | 62,533 |
| #22 | TITLE-ABS-KEY ( "Oral health related quality of life" ) | 3,693 |
| #21 | TITLE-ABS-KEY ( ohrqol ) | 2,143 |
| #20 | TITLE-ABS-KEY ( hrqol ) | 23,334 |
| #19 | TITLE-ABS-KEY ( "Quality of life*" ) | 651,371 |
| #18  #17  #16 | TITLE-ABS-KEY ( qol )  TITLE-ABS-KEY ( "european organisation for research and treatment of cancer" )  TITLE-ABS-KEY ( "quality of life assessment" ) | 58,588  7634  15616 |
| #15 | TITLE-ABS-KEY ( "Mouth rehabilitation" ) | 1,953 |
| #14 | TITLE-ABS-KEY ( "Dental rehabilitation" ) | 822 |
| #13 | TITLE-ABS-KEY ( "Implant overdenture*" ) | 740 |
| #12 | TITLE-ABS-KEY ( "implant borne*" ) | 208 |
| #11  #10  #9  #8  #7  #6  #5  #4  #3  #2  #1 | TITLE-ABS-KEY ( "implant retained*" )  TITLE-ABS-KEY ( "Implant supported*")  TITLE-ABS-KEY ( "Dental implant*" )  TITLE-ABS-KEY ( "dental prosthesis, implant-supported" )  TITLE-ABS-KEY ( "full mouth rehabilitation" )  TITLE-ABS-KEY ( "endosseous implant" )  TITLE-ABS-KEY ( "tooth implant" )  TITLE-ABS-KEY ( "mandibular prosthe*" )  TITLE-ABS-KEY ( "fixed denture*" )  TITLE-ABS-KEY ( "Implant denture*" )  TITLE-ABS-KEY ( "Oral rehabilitation" ) | 1,537  12,697  52,125  9255  583  1849  16261  1145  537  204  2050 |
|  |  |  |
|  |  |  |

**Web of Science Core Collection (updated 5/1/2024)**

| #41 | #39 AND #16 and English (Languages) | 1840 |
| --- | --- | --- |
| #40 | #39 AND #16 | 1960 |
| #39 | #38 OR #37 OR #36 OR #35 OR #34 OR #33 OR #32 OR #31 OR #30 OR #29 OR #28 OR #27 OR #26 OR #25 OR #24 OR #23 OR #22 OR #21 OR #20 OR #19 OR #18 OR #17 | 680,446 |
| #38 | TS=(WHOQOL) | 5,445 |
| #37 | TS=(EuroQol) | 9,469 |
| #36 | TS=("VAS scal*") | 1,215 |
| #35 | TS=(EORTC*) | 14,058 |
| #34 | TS=(UWQ*) | 77 |
| #33 | TS=("University of Washington*") | 5,082 |
| #32 | TS=(OHIP*) | 2,280 |
| #31 | TS=("Oral Health Impact Profile*") | 2,043 |
| #30 | TS=("Self reported outcom*") | 1,365 |
| #29 | TS=("Functional outcome*") | 65,440 |
| #28 | TS=("Patient reported outcome*") | 46,690 |
| #27 | TS=("Patient reported outcome measure*") | 13,955 |
| #26 | TS=(PROMs) | 10,378 |
| #25 | TS=("Validated tool*") | 4,247 |
| #24 | TS=("Health related quality of life") | 70,367 |
| #23 | TS=("Oral health related quality of life") | 3651 |
| #22 | TS=(OHRQOL) | 2166 |
| #21 | TS=(HRQOL) | 24,878 |
| #20 | TS=("Quality of life*") | 571,483 |
| #19 | TS=(QOL) | 57,181 |
| #18 | TS=("european organisation for research and treatment of cancer") | 1887 |
| #17 | TS=("quality of life assessment") | 3,695 |
| #16 | #15 OR #14 OR #13 OR #12 OR #11 OR #10 OR #9 OR #8 OR #7 OR #6 OR #5 OR #4 OR #3 OR #2 OR #1 | 37,116 |
| #15 | TS=("Implant retained*") | 1,203 |
| #14 | TS=("Implant supported*") | 5,801 |
| #13 | TS=("Dental implant*") | 30,907 |
| #12 | TS=("Implant overdenture*") | 732 |
| #11 | TS=("dental prosthesis, implant-supported" ) | 24 |
| #10 | TS=("full mouth rehabilitation" ) | 266 |
| #9 | TS=("endosseous implant") | 408 |
| #8 | TS=("tooth implant" ) | 631 |
| #7 | TS=("mandibular prosthe*") | 305 |
| #6 | TS=("fixed denture*") | 226 |
| #5 | TS=( "Implant denture*") | 73 |
| #4 | TS=("Implant borne*") | 155 |
| #3 | TS=("dental rehabilitation") | 696 |
| #2 | TS=("mouth rehabilitation") | 378 |
| #1 | TS=("Oral rehabilitation") | 1725 |

**Handle on Qol: head and neck database listing evidence on QOL (updated 5/1/2024)**

| Dental implant | 29 |
| --- | --- |
| Implant overdenture | 0 |
| dental prosthesis, implant-supported | 1 |
| full mouth rehabilitation | 0 |
| endosseous implant | 0 |
| tooth implant | 0 |
| mandibular prosthe* | 3 |
| fixed denture* | 1 |
| Implant denture* | 0 |
| Implant borne* | 2 |
| dental rehabilitation | 19 |
| mouth rehabilitation | 2 |
| Oral rehabilitation | 44 |
| Implant retained* | 13 |
| Implant supported* | 13 |

**Total = 127**

**Searching of reference list: 25**

**Appendix 1b: COSMIN Content Validity Search Strategy**

**Pubmed (Searched 11/8/2023)**

**Construct**

#1

"health related quality of life"[Title/Abstract] OR "oral health related quality of life"[Title/Abstract] OR "ohrqol"[Title/Abstract] OR "hrqol"[Title/Abstract] OR "quality of life"[MeSH Terms] OR "quality of life*"[Title/Abstract] OR "qol"[Title/Abstract] OR "quality of life assessment"[Title/Abstract]) AND "humans"[MeSH Terms]

**Population**

#2

(("implant denture*"[Title/Abstract] OR "Oral rehabilitation"[Title/Abstract] OR "Mouth rehabilitation"[MeSH Terms] OR "Mouth rehabilitation"[Title/Abstract] OR "Dental rehabilitation"[Title/Abstract] OR "implant overdenture*"[Title/Abstract] OR "implant borne*"[Title/Abstract] OR "implant retained*"[Title/Abstract] OR "implant supported*"[Title/Abstract] OR "dental implants"[MeSH Terms] OR "dental prosthesis, implant supported"[MeSH Terms] OR "full mouth rehabilitation"[Title/Abstract] OR "mandibular prosthe*"[Title/Abstract] OR ("mandibular prosthesis"[MeSH Terms] OR "mandibular prosthesis implantation"[MeSH Terms]) OR "fixed denture*"[Title/Abstract] OR "dental implantation"[MeSH Terms] OR "Mandibular Reconstruction"[MeSH Terms] OR "Mandibular Reconstruction"[Title/Abstract] OR "Maxilla Reconstruction"[Title/Abstract] OR "surgical flaps"[MeSH Terms] OR "free flap*"[Title/Abstract] OR "jaw cysts"[MeSH Terms] OR "jaw cyst*"[Title/Abstract] OR "odontogenic tumors"[MeSH Terms] OR "oral tumo*"[Title/Abstract] OR "Osteoradionecrosis"[MeSH Terms] OR "Osteoradionecrosis"[Title/Abstract] OR "jaw neoplasms"[MeSH Terms] OR "jaw neoplasms"[TIAB] OR "mouth neoplasm*"[Title/Abstract] OR "oral malignanc*"[Title/Abstract] OR "oral cancer"[Title/Abstract] OR "Maxillectomy"[Title/Abstract] OR "mandibulectomy"[Title/Abstract]) AND "humans"[MeSH Terms])

**Instrument types**

#3

“european organisation for research and treatment of cancer”[TIAB] OR “eortc*”[TIAB] OR “University of Washington*”[TIAB] OR “UWQ*”[TIAB] OR “UW-QOL*”[TIAB] OR “Oral Health Impact Profile”[TIAB] OR “OHIP*”[TIAB]

**Measurement properties**

#4

(instrumentation[sh] OR methods[sh] OR "Validation Studies"[pt] OR "Comparative Study"[pt] OR "psychometrics"[MeSH] OR psychometr*[tiab] OR clinimetr*[tw] OR clinometr*[tw] OR "outcome assessment (health care)"[MeSH] OR "outcome assessment"[tiab] OR "outcome measure*"[tw] OR "observer variation"[MeSH] OR "observer variation"[tiab] OR "Health Status Indicators"[Mesh] OR "reproducibility of results"[MeSH] OR reproducib*[tiab] OR "discriminant analysis"[MeSH] OR reliab*[tiab] OR unreliab*[tiab] OR valid*[tiab] OR "coefficient of variation"[tiab] OR coefficient[tiab] OR homogeneity[tiab] OR homogeneous[tiab] OR "internal consistency"[tiab] OR (cronbach*[tiab] AND (alpha[tiab] OR alphas[tiab])) OR (item[tiab] AND (correlation*[tiab] OR selection*[tiab] OR reduction*[tiab])) OR agreement[tw] OR precision[tw] OR imprecision[tw] OR "precise values"[tw] OR test-retest[tiab] OR (test[tiab] AND retest[tiab]) OR (reliab*[tiab] AND (test[tiab] OR retest[tiab])) OR stability[tiab] OR interrater[tiab] OR inter-rater[tiab] OR intrarater[tiab] OR intra-rater[tiab] OR intertester[tiab] OR inter-tester[tiab] OR intratester[tiab] OR intra-tester[tiab] OR interobserver[tiab] OR inter-observer[tiab] OR intraobserver[tiab] OR intra-observer[tiab] OR intertechnician[tiab] OR inter-technician[tiab] OR intratechnician[tiab] OR intra-technician[tiab] OR interexaminer[tiab] OR inter-examiner[tiab] OR intraexaminer[tiab] OR intra-examiner[tiab] OR interassay[tiab] OR inter-assay[tiab] OR intraassay[tiab] OR intra-assay[tiab] OR interindividual[tiab] OR inter-individual[tiab] OR intraindividual[tiab] OR intra-individual[tiab] OR interparticipant[tiab] OR inter-participant[tiab] OR intraparticipant[tiab] OR intra-participant[tiab] OR kappa[tiab] OR kappa's[tiab] OR kappas[tiab] OR repeatab*[tw] OR ((replicab*[tw] OR repeated[tw]) AND (measure[tw] OR measures[tw] OR findings[tw] OR result[tw] OR results[tw] OR test[tw] OR tests[tw])) OR generaliza*[tiab] OR generalisa*[tiab] OR concordance[tiab] OR (intraclass[tiab] AND correlation*[tiab]) OR discriminative[tiab] OR "known group"[tiab] OR "factor analysis"[tiab] OR "factor analyses"[tiab] OR "factor structure"[tiab] OR "factor structures"[tiab] OR dimension*[tiab] OR subscale*[tiab] OR (multitrait[tiab] AND scaling[tiab] AND (analysis[tiab] OR analyses[tiab])) OR "item discriminant"[tiab] OR "interscale correlation*"[tiab] OR error[tiab] OR errors[tiab] OR "individual variability"[tiab] OR "interval variability"[tiab] OR "rate variability"[tiab] OR (variability[tiab] AND (analysis[tiab] OR values[tiab])) OR (uncertainty[tiab] AND (measurement[tiab] OR measuring[tiab])) OR "standard error of measurement"[tiab] OR sensitiv*[tiab] OR responsive*[tiab] OR (limit[tiab] AND detection[tiab]) OR "minimal detectable concentration"[tiab] OR interpretab*[tiab] OR ((minimal[tiab] OR minimally[tiab] OR clinical[tiab] OR clinically[tiab]) AND (important[tiab] OR significant[tiab] OR detectable[tiab]) AND (change[tiab] OR difference[tiab])) OR (small*[tiab] AND (real[tiab] OR detectable[tiab]) AND (change[tiab] OR difference[tiab])) OR "meaningful change"[tiab] OR "ceiling effect"[tiab] OR "floor effect"[tiab] OR "Item response model"[tiab] OR IRT[tiab] OR Rasch[tiab] OR "Differential item functioning"[tiab] OR DIF[tiab] OR "computer adaptive testing"[tiab] OR "item bank"[tiab] OR "cross-cultural equivalence"[tiab])

NOT #5

("addresses"[Publication Type] OR "biography"[Publication Type] OR "case reports"[Publication Type] OR "comment"[Publication Type] OR "directory"[Publication Type] OR "editorial"[Publication Type] OR "festschrift"[Publication Type] OR "interview"[Publication Type] OR "lectures"[Publication Type] OR "legal cases"[Publication Type] OR "legislation"[Publication Type] OR "letter"[Publication Type] OR "news"[Publication Type] OR "newspaper article"[Publication Type] OR "patient education handout"[Publication Type] OR "popular works"[Publication Type] OR "congresses"[Publication Type] OR "consensus development conference"[Publication Type] OR "consensus development conference, nih"[Publication Type] OR "practice guideline"[Publication Type]) NOT ("animals"[MeSH Terms] NOT "humans"[MeSH Terms])

#1 AND #2 AND #3 AND #4 NOT #5 filters human and English = 447

**Embase (Searched 11/8/2023)**

**Construct**

#1

'health related quality of life':ab,ti OR 'oral health related quality of life':ab,ti OR 'ohrqol':ab,ti OR 'hrqol':ab,ti OR 'quality of life'/exp OR 'quality of life*':ab,ti OR 'qol':ab,ti OR 'quality of life assessment'/exp

**Population**

# 2

"implant denture*":ab,ti OR "oral rehabilitation":ab,ti OR "full mouth rehabilitation"/exp OR "mouth rehabilitation":ab,ti OR "dental rehabilitation":ab,ti OR "implant overdenture*":ab,ti OR "implant borne*":ab,ti OR "implant retained*":ab,ti OR "implant supported*":ab,ti OR "dental implants":ab,ti OR "implant supported denture"/exp OR "full mouth rehabilitation":ab,ti OR "mandibular prosthe*":ab,ti OR "tooth implant"/exp OR "fixed denture*":ab,ti OR "endosseous implant":ab,ti OR "endosseous implant"/exp OR "mandibulectomy"/exp OR "mandibulectomy":ab,ti OR "mandible reconstruction"/exp OR "mandible reconstruction":ab,ti OR "maxilla resection”/exp OR "maxilla reconstruction":ab,ti OR "surgical flaps"/exp OR "free flap*":ab,ti OR "jaw tumor"/exp OR "jaw tumo*" OR "osteoradionecrosis"/exp OR "osteoradionecrosis":ab,ti OR "mouth tumor"/exp OR "mouth tumor*":ab,ti OR "microvascular surgery"/exp OR "oral malignanc*":ab,ti OR "oral cancer":ab,ti OR "maxillectomy":ab,ti

**Instrument types**

#3

“european organization for research and treatment of cancer quality of life questionnaire core 30”/exp OR “european organisation for research and treatment of cancer”:ab,ti OR “eortc*”:ab,ti OR “University of Washington*”:ab,ti OR “UWQ*”:ab,ti OR “UW-QOL*”:ab,ti OR “Oral Health Impact Profile”:ab,ti OR “OHIP*”

**Measurement properties**

#4

'intermethod comparison'/exp OR 'data collection method'/exp OR 'validation study'/exp OR 'feasibility study'/exp OR 'pilot study'/exp OR 'psychometry'/exp OR 'reproducibility'/exp OR reproducib*:ab,ti OR 'audit':ab,ti OR psychometr*:ab,ti OR clinimetr*:ab,ti OR clinometr*:ab,ti OR 'observer variation'/exp OR 'observer variation':ab,ti OR 'discriminant analysis'/exp OR 'validity'/exp OR reliab*:ab,ti OR valid*:ab,ti OR 'coefficient':ab,ti OR 'internal consistency':ab,ti OR (cronbach*:ab,ti AND ('alpha':ab,ti OR 'alphas':ab,ti)) OR 'item correlation':ab,ti OR 'item correlations':ab,ti OR 'item selection':ab,ti OR 'item selections':ab,ti OR 'item reduction':ab,ti OR 'item reductions':ab,ti OR 'agreement':ab,ti OR 'precision':ab,ti OR 'imprecision':ab,ti OR 'precise values':ab,ti OR 'test-retest':ab,ti OR ('test':ab,ti AND 'retest':ab,ti) OR (reliab*:ab,ti AND ('test':ab,ti OR 'retest':ab,ti)) OR 'stability':ab,ti OR 'interrater':ab,ti OR 'inter-rater':ab,ti OR 'intrarater':ab,ti OR 'intra-rater':ab,ti OR 'intertester':ab,ti OR 'inter-tester':ab,ti OR 'intratester':ab,ti OR 'intra-tester':ab,ti OR 'interobeserver':ab,ti OR 'inter-observer':ab,ti OR 'intraobserver':ab,ti OR 'intra-observer':ab,ti OR 'intertechnician':ab,ti OR 'inter-technician':ab,ti OR 'intratechnician':ab,ti OR 'intra-technician':ab,ti OR 'interexaminer':ab,ti OR 'inter-examiner':ab,ti OR 'intraexaminer':ab,ti OR 'intra-examiner':ab,ti OR 'interassay':ab,ti OR 'inter-assay':ab,ti OR 'intraassay':ab,ti OR 'intra-assay':ab,ti OR 'interindividual':ab,ti OR 'inter-individual':ab,ti OR 'intraindividual':ab,ti OR 'intra-individual':ab,ti OR 'interparticipant':ab,ti OR 'inter-participant':ab,ti OR 'intraparticipant':ab,ti OR 'intra-participant':ab,ti OR 'kappa':ab,ti OR 'kappas':ab,ti OR 'coefficient of variation':ab,ti OR repeatab*:ab,ti OR (replicab*:ab,ti OR 'repeated':ab,ti AND ('measure':ab,ti OR 'measures':ab,ti OR 'findings':ab,ti OR 'result':ab,ti OR 'results':ab,ti OR 'test':ab,ti OR 'tests':ab,ti)) OR generaliza*:ab,ti OR generalisa*:ab,ti OR 'concordance':ab,ti OR ('intraclass':ab,ti AND correlation*:ab,ti) OR 'discriminative':ab,ti OR 'known group':ab,ti OR 'factor analysis':ab,ti OR 'factor analyses':ab,ti OR 'factor structure':ab,ti OR 'factor structures':ab,ti OR 'dimensionality':ab,ti OR subscale*:ab,ti OR 'multitrait scaling analysis':ab,ti OR 'multitrait scaling analyses':ab,ti OR 'item discriminant':ab,ti OR 'interscale correlation':ab,ti OR 'interscale correlations':ab,ti OR ('error':ab,ti OR 'errors':ab,ti AND (measure*:ab,ti OR correlat*:ab,ti OR evaluat*:ab,ti OR 'accuracy':ab,ti OR 'accurate':ab,ti OR 'precision':ab,ti OR 'mean':ab,ti)) OR 'individual variability':ab,ti OR 'interval variability':ab,ti OR 'rate variability':ab,ti OR 'variability analysis':ab,ti OR ('uncertainty':ab,ti AND ('measurement':ab,ti OR 'measuring':ab,ti)) OR 'standard error of measurement':ab,ti OR sensitiv*:ab,ti OR responsive*:ab,ti OR ('limit':ab,ti AND 'detection':ab,ti) OR 'minimal detectable concentration':ab,ti OR interpretab*:ab,ti OR (small*:ab,ti AND ('real':ab,ti OR 'detectable':ab,ti) AND ('change':ab,ti OR 'difference':ab,ti)) OR 'meaningful change':ab,ti OR 'minimal important change':ab,ti OR 'minimal important difference':ab,ti OR 'minimally important change':ab,ti OR 'minimally important difference':ab,ti OR 'minimal detectable change':ab,ti OR 'minimal detectable difference':ab,ti OR 'minimally detectable change':ab,ti OR 'minimally detectable difference':ab,ti OR 'minimal real change':ab,ti OR 'minimal real difference':ab,ti OR 'minimally real change':ab,ti OR 'minimally real difference':ab,ti OR 'ceiling effect':ab,ti OR 'floor effect':ab,ti OR 'item response model':ab,ti OR 'irt':ab,ti OR 'rasch':ab,ti OR 'differential item functioning':ab,ti OR 'dif':ab,ti OR 'computer adaptive testing':ab,ti OR 'item bank':ab,ti OR 'cross-cultural equivalence':ab,ti AND [embase]/lim

#1 AND #2 AND #3 AND #4 with filters 'human'/de AND [english]/lim AND 'article'/it OR 'article in press'/it OR 'conference paper'/it) = 439
